# Whole genome sequencing and phylogenetic analysis of human metapneumovirus strains from Kenya and Zambia

**DOI:** 10.1186/s12864-019-6400-z

**Published:** 2020-01-02

**Authors:** Everlyn Kamau, John W. Oketch, Zaydah R. de Laurent, My V. T. Phan, Charles N. Agoti, D. James Nokes, Matthew Cotten

**Affiliations:** 1 KEMRI-Wellcome Trust Research Programme, Kilifi, Kenya; 2 Department of Viroscience, Erasmus MC, Rotterdam, The Netherlands; 3 School of Life Sciences and Zeeman Institute, University of Warwick, Coventry, UK; 4 MRC/UVRI & LSHTM Uganda Research Unit, Entebbe, Uganda; 5 MRC-University of Glasgow Centre for Virus Research, Glasgow, UK

**Keywords:** Metapneumovirus, Genome, Diversity

## Abstract

**Background:**

Human metapneumovirus (HMPV) is an important cause of acute respiratory illness in young children. Whole genome sequencing enables better identification of transmission events and outbreaks, which is not always possible with sub-genomic sequences.

**Results:**

We report a 2-reaction amplicon-based next generation sequencing method to determine the complete genome sequences of five HMPV strains, representing three subgroups (A2, B1 and B2), directly from clinical samples. In addition to reporting five novel HMPV genomes from Africa we examined genetic diversity and sequence patterns of publicly available HMPV genomes. We found that the overall nucleotide sequence identity was 71.3 and 80% for HMPV group A and B, respectively, the diversity between HMPV groups was greater at amino acid level for SH and G surface protein genes, and multiple subgroups co-circulated in various countries. Comparison of sequences between HMPV groups revealed variability in G protein length (219 to 241 amino acids) due to changes in the stop codon position. Genome-wide phylogenetic analysis showed congruence with the individual gene sequence sets except for F and M2 genes.

**Conclusion:**

This is the first genomic characterization of HMPV genomes from African patients.

## Background

Human metapneumovirus (HMPV) is a single-stranded RNA virus in the family *Paramyxoviridae* and closely related to human respiratory syncytial virus (RSV) [1]. HMPV causes respiratory disease similar to RSV, ranging from mild upper respiratory infection to bronchiolitis and pneumonia [2]. HMPV infections are seasonal and co-infection with other respiratory pathogens is common [1]. The HMPV genome is approximately 13 kb and comprises eight open reading frames (ORFs) encoding nucleoprotein (N), phosphoprotein (P), matrix protein (M), fusion glycoprotein (F), transcription enhancer protein (M2), small hydrophobic protein (SH), attachment glycoprotein (G), and large polymerase protein (L) [3]. The membrane glycoproteins F and G sequences are used to define two major genotypes or groups, A and B, which are further classified into four subgroups (A1, A2, B1, and B2). HMPV A2, the most frequently observed subgroup, is further divided into two proposed sub-lineages (A2a and A2b) [3].

HMPV is reported to have an important contribution to acute respiratory infections (ARI) in Africa. For instance, HMPV-associated hospitalization was estimated at 6.5 per 1000 person years in infants in Soweto, South Africa [4]; at 4% in hospitalized children with severe ARI during a 2-year period in Cameroon [5]; and in rural western Kenya, incidence of HMPV associated with ARI cases in outpatient clinic visits was estimated at 0.43 per 100 person-years among outpatients [6]. In Kilifi coastal Kenya, between January 2007 to December 2011, children under 6 months of age accounted for 44% of HMPV positive cases, while 74% were children under 1 year, and 1.3% (2/160) were children > 36 months [7]. In Dadaab and Kakuma refugee camps in Kenya, HMPV was detected in 5.7% hospitalizations, and virus-positive crude hospitalization rate (per 1000 children < 5 years old) was 4 for HMPV [8]. In Mali, contribution of HMPV to pneumonia had a population attributable fraction of 9% (95% CI: 7–11%) [9]; while in Morocco [10], 8.9% of children < 5 years admitted with severe pneumonia were infected with HMPV. HMPV prevalence and incidence elsewhere globally, is indicated in Additional file 4: Table S1. Of note is that the variations in incidence rates could be attributed to study population, seasonality and even detection methods. Nonetheless, genomic epidemiology of HMPV in Africa is inadequately reported, and comparison of genetic similarity and differences between African and global strains is not documented.

Genome sequences provide valuable resources for characterizing viral evolution and disease epidemiology, and for identifying transmission events and outbreaks, which is not always possible with sub-genomic fragments [11–13]. The increased number of phylogenetically informative variant sites obtained from full genomes may allow better linking of cases and aid public health interventions in real time during epidemics [14, 15]. PCR approaches for targeted whole genome sequencing, in contrast to random amplification, can preferentially amplify the target virus over host or environmental nucleic acids [16, 17] potentially focusing sequencing on the virus of interest. To date, the largest dataset of HMPV whole genomes (*n = 61*) sequenced from any tropical country is from three Peruvian cities, Lima, Piura and Iquitos [18]. In Africa, apart from one metapneumovirus genome identified from a wild mountain gorilla in Rwanda (GenBank accession number HM197719), there are no HMPV genomes reported according to the NIAID Virus Pathogen Database and Analysis Resource (ViPR, http://www.viprbrc.org/, accessed April 30, 2019). This has led to limited understanding of the genetic and genomic diversity of HMPV in the continent.

This work describes a whole genome sequencing (WGS) approach for HMPV from a small number of HMPV positive clinical samples collected at Kilifi County Hospital in Kilifi, Kenya and University Teaching Hospital in Lusaka, Zambia. The genomes were generated by sequencing overlapping PCR amplicons spanning the entire genome. These are the first reported complete genome sequences of locally circulating HMPV strains obtained directly from clinical samples in Africa. We also combined the new genomes with publicly available sequences to examine patterns in global HMPV genetic diversity.

## Results

### Genome characteristics

Whole genome sequencing was successful for all 5 clinical samples that were attempted. A single genomic sequence was obtained from each sample, and the length of the 5 new HMPV genomes ranged from 13,097 to 13,134 nt (> 95% length coverage). Sequencing and data assembly parameters, including coverage depth are shown in Table 1.

**Table 1:** Sequencing results and data assembly metrics for the five HMPV genomes. Data assembly parameters are provided in the table footnotes

Strain	Total reads	HMPV reads^a^	N50^b^	Genome length	Ct^c^	Mean coverage^d^	GenBank accession	Sub-group^e^
HMPV/01/KEN/2015	916,378	42%	12,561	13,101	27.4	14,430.68	MK588634	A2b
HMPV/02/KEN/2012	653,642	38%	13,032	13,104	26.0	4856.963	MK588637	B2
HMPV/03/KEN/2013	684,104	39%	12,962	13,134	21.0	7207.968	MK588633	A2b
HMPV/04/KEN/2012	766,948	22%	12,489	13,097	22	6764.68	MK588636	B1
HMPV/05/ZAM/2012	962,668	26%	13,011	13,145	27.2	10,444.95	MK588635	A2a

^a^Percentage of sequencing reads classified as HMPV

^b^N50 length (a measure of genome assembly completeness) is calculated by summing lengths of assembled contigs from the longest to the shortest and determining the point at which 50% of the total assembly size is reached.

^c^rRT-PCR cycle threshold value.

^d^Calculated by dividing the per-position coverage output (described in Methods) by respective genome length

^e^As assigned by phylogenetic analyses.

Sequence annotation of the full-length genomes using Geneious R8.1.5 (https://www.geneious.com) identified the expected eight coding ORFs and non-coding genomic regions. The overall nucleotide identity (i.e., identical sites averaging over all sequence pairs and excluding positions containing gaps) between all 143 genome sequences analyzed (5 new genomes plus 138 from ViPR) was 58.2%. Nucleotide sequence identity was 71.3% within HMPV-A and 80% within HMPV-B. Intra-subgroup, A1, A2, B1 and B2 genomes shared 92.1% (10 sequences), 76.8% (88 sequences), 91% (24 sequences) and 89.6% (21 sequences) amino acid sequence identity.

**Fig. 1:**
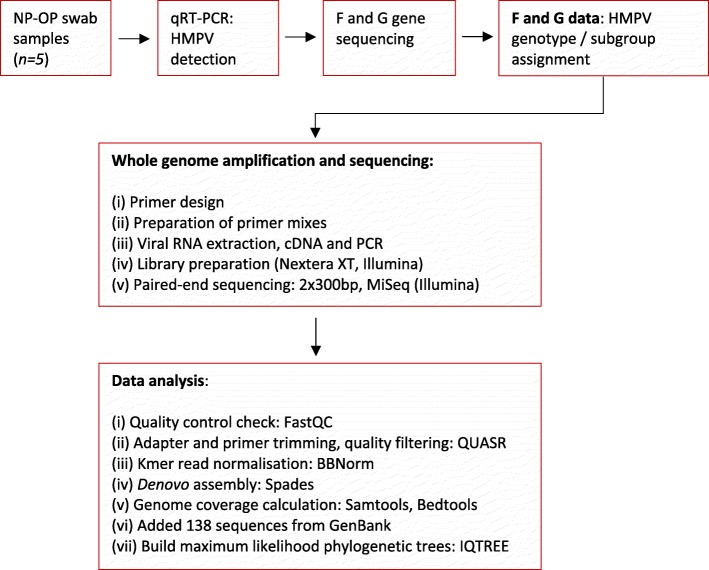
Consensus nucleotide sequences of putative gene-start (13 nucleotides upstream of ATG codon) and gene-end signals (6–16 nucleotides from Stop codon) visualized as sequence logos, for HMPV group (**a**) and (**b**). The height of each character in the sequence logo plots is proportional to its relative frequency. The green color on the bar at the bottom of the consensus sequence logo indicates 100% average pairwise identity, brown indicates at least 30 to < 100% identity and red indicates < 30% identity

For the 143 HMPV genomes, we checked sequence conservation at transcriptional control regions, at the termini of each gene, as well as the lengths of intergenic sequences between gene boundaries. The length of the F-M2 intergenic region was different between group A and B viruses, that is, 13 nt and 2 nt, respectively. The SH-G and G-L intergenic regions were the longest, up to 125 nt and to 190 nt, respectively. Consensus nucleotides (9 to 19 length) at the putative start and end regions flanking the ORF of the viral genes are shown in Fig. 1.

The gene-start and -end regions of N and P were conserved (> 90% average pairwise identity) in both HMPV groups, and the M2 and M gene-start and -end were also conserved in HMPV group A and B, respectively. The putative ATG start codon was consistently located at positions 14–16 upstream of a gene start motif (consensus: GG/AGAC/TAAA/GTnnnnATG), except for the internal M2–2. An additional ATG start codon upstream of the gene-start motif was observed in the SH gene for the B1 and B2 strains. In five of the eight annotated genes (N, P, F, M2, and G (B1 and B2 strains only)), the intergenic regions were short and the ORFs for these 5 genes terminated within the propositioned gene-end motifs.

### Sequence diversity and phylogenetic relationships

We combined the five genome sequences from Kenya and Zambia with available global sequences, aligned individual genes and calculated the percent nucleotide (nt) and amino acid (aa) identity (Table 2).

**Table 2:** Levels of nucleotide and amino acid identity^a^ of sequences within the two major HMPV groups. This analysis included the new sequences from Kenya and Zambia plus the global sequences retrieved from GenBank

	Average % nucleotide identity		Average % amino acid identity	
	Group A	Group B	Group A	Group B
N	90.3	91.3	97.6	98.4
P	86.6	87.4	90.0	91.1
M	90.1	90.7	97.8	98.5
F	88.6	88.9	94.8	96.0
M2	89.0	89.9	94.2	95.7
SH	75.8	76.5	72.5	73.8
G	63.0	66.9	50.1	57.8
L	89.8	90.3	96.5	97.6

^a^Calculated as mean pairwise identity over all pairs in a column or position.

**Fig. 2:**
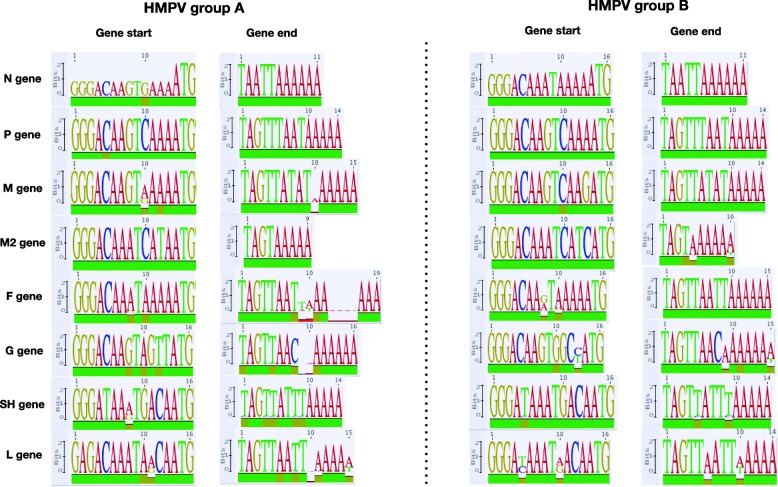
Average pairwise identity over all pairs in an alignment for every position of the predicted G glycoprotein amino acid sequences, for HMPV groups (**a**) and (**b**). The dataset analyzed here included all available genomes (Kenya and Zambia (*n* = 5) plus 138 from other locations globally). The average pairwise identities were calculated in Geneious R8.1.5. Black bars indicate > 50% (> 0.5) average amino acid identity and red bars indicate < 50% (< 0.5) non-identity among sequences. Proposed intracellular (positions 1 to 32), transmembrane (TM, positions 33 to 51), and extracellular (positions 52 to 220 for group (**a**), 52 to 242 for group (**b**) domains are indicated above the plots

**Fig. 3:**
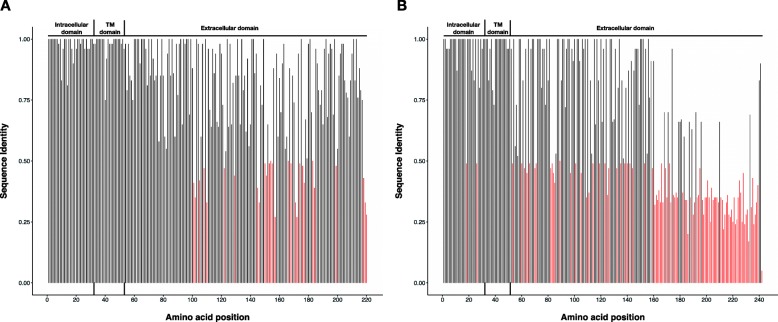
Mid-pointed maximum-likelihood (ML) phylogenetic trees of SH glycoprotein gene (**a**) G glycoprotein gene (**b**) and the full-length genome sequences (**c**) of viruses from Kenya and Zambia (marked in red), plus 138 other sequences (> 13 kb) retrieved from GenBank (Additional Table 3). Bootstrap support values (evaluated by 1000 replicates) are indicated along the branches. Genetic subgroups A1, A2a, A2b, B1, and B2, are indicated. Multiple sequence alignment was done using MAFFT and the ML phylogeny inferred using GTR + Γ nucleotide substitution model and ultrafast bootstrap approximation in IQ-TREE. The genotype B2 Sabana strain sequence (GenBank accession number HM197719) reported from a wild mountain gorilla in Rwanda is marked in blue. The scaled bar indicates nucleotide substitutions per site

The coding sequences of N, M, F, M2–1, M2–2, and L genes were conserved at nucleotide and amino acid levels, by sharing > 85% between-subgroup nucleotide identity and 90% protein identity (Table 3). The nucleoprotein gene was the most conserved among all subgroups at the nt and aa levels. SH and G glycoprotein genes were more divergent between the HMPV subgroups at the nucleotide level with 76 and 63% identity, respectively. The SH protein length was variable between group A and B strains due to a nucleotide substitution (CAA ➔ TAA) at gene position 532 in group B, resulting in protein lengths of 178 and 180 aa, respectively. The predicted G protein length also varied among the different HMPV subgroups, between 219 and 241 aa, due to different positions of the Stop codon. Amino acid sequence diversity for G and SH glycoproteins is depicted in Fig. 2 and Additional file 2: Figure S2, respectively. The diversity of the complete nucleotide sequences of SH and G genes is depicted in phylogenetic trees in Fig. 3.

**Table 3:** Demographic and clinical information collected from patients at the point of sampling

Strain ID	Sample collection date	Gender	Age	Clinical manifestation
HMPV/01/KEN/2015	23/12/2015	Male	5 months	Fever, cough, difficulty breathing, severe pneumonia
HMPV/02/KEN/2012	15/04/2012	Female	7 months	Fever, cough, chest indrawing, severe pneumonia
HMPV/03/KEN/2013	30/10/2012	Male	2 months	Cough, breathing difficulty, chest indrawing, crackles, severe pneumonia
HMPV/04/KEN/2012	06/01/2012	Male	4 years	Fever, breathing difficulty, vomiting, severe pneumonia
HMPV/05/ZAM/2012	28/02/2012	Female	7 months	Fever, cough, difficulty breathing, severe pneumonia

We evaluated phylogenetic classification and relationship between the 5 new genomes obtained in this study and previously published genomes (Fig. 3). Full genome classification was consistent with that based on partial genomic fragments (F and G genes). Two genomes from the samples collected in Kenya (HMPV/03/KEN/2013) and (HMPV/01/KEN/2015), clustered closely to viruses from USA (collected in 2016/17) and Thailand (collected in 2013) within the A2 subgroup (Fig. 3). The A2a virus sequenced from Zambia (HMPV/05/ZAM/2012) clustered closely with Peruvian HMPV strains collected in 2012, with > 89% nt similarity, and the B2 genome from Kenya (strain HMPV/02/KEN/2012) shared more than 90% nt similarity with a virus collected in the U.S. in 2015. The B1 genome from Kenya (strain HMPV/04/KEN/2012) exhibited 97.5% nucleotide similarity with viruses collected in 2004 in Australia and USA. The diversity of the complete nucleotide sequences of N, P, M, F, M2 and L genes is depicted in phylogenetic trees in Additional file 3: Figure S3. There was phylogenetic congruence with the individual gene sequence sets as with the full genome dataset, except for F and M2 gene (Additional file 3: Figure S3).

### Sequence diversity at rRT-PCR target region

**Fig. 4:**
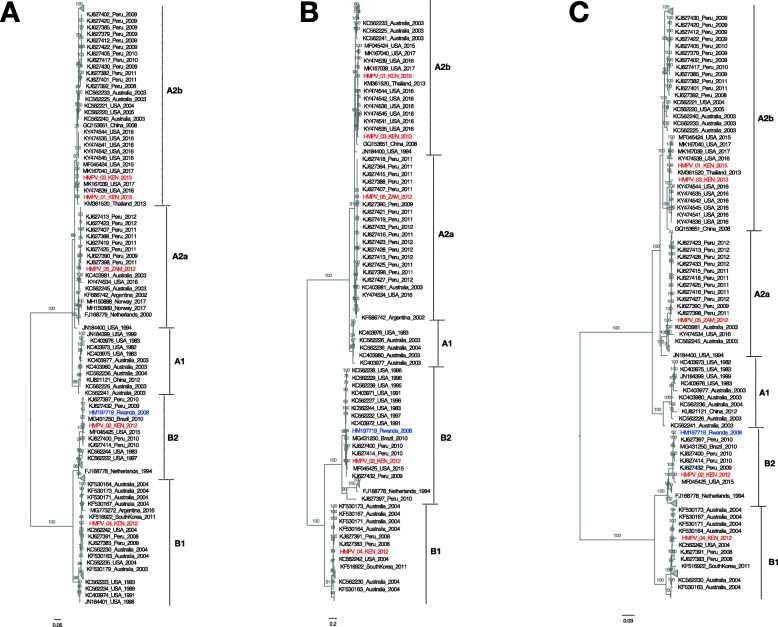
Mismatches between the rRT-PCR diagnostic primers and probes and their expected binding sites in the five genomes from Kenya and Zambia. ‘Fwd primer’ = Forward primer and ‘Rev primer’ = Reverse primer. Two rRT-PCR assays were used for HMPV detection. The colored bars in the figure indicate nucleotide differences (mismatches) between (**a**) three HMPV-A genomes and HMPV-A specific primers and probes targeting fusion gene, (**b**) two HMPV-B genomes and HMPV-B specific primers and probes also targeting fusion gene, and (**c**) all five genomes reported here and specific primers and probes targeting nucleoprotein gene. The sequences of the rRT-PCR primers and probes checked against the African HMPV genomes are listed in Additional file 7: Table S4

Variant or drifted viral strains may lower the sensitivity of detection resulting in a decreased quantitation of the viral load and underestimation of disease incidence [19]. We checked the new HMPV genomes for nucleotide differences in the genomic regions targeted by our diagnostic rRT-PCR primers and probes (Additional file 7: Table S4) used for HMPV detection. Up to eight primer- and probe-template mismatches were identified (Fig. 4): one mismatch in the forward primer region in HMPV group A (F gene-based rRT-PCR assay, Fig. 4a); one mismatch in each of the forward and probe target regions in group B (F gene-based rRT-PCR assay, Fig. 4b); and 5 different mismatches with the N-gene based rRT-PCR assay (Fig. 4c). Note, the F gene-based rRT-PCR assays are different or specific to the two HMPV groups.

## Discussion

HMPV causes respiratory illness presenting as mild upper respiratory tract infection or life-threatening severe bronchiolitis and pneumonia primarily in children, sometimes adults as well as immunocompromised individuals [2]. However, HMPV genome sequence data from Africa is sparse and information on genome-wide diversity is limited. In the present study, the whole genome sequences of five HMPV strains from Kenya and Zambia were determined and compared with the genomes published previously from around the world. Comparative sequence analysis indicated fairly conserved positioning of the gene-start and -end regions as well as translational start and -end codons. Variation in gene-start and -end sequences can have significant impact on transcription initiation and termination efficiency so that there is more selective pressure preventing changes in these regions [20], and this likely explains our observation. The additional ATG start codon found upstream of the gene-start motif of the SH gene was consistent with a previous report [21], though its role in gene expression is yet to be identified.

These observed sequence conservation in N, M, F, M2–1, M2–2, and L genes is not unusual and is suggestive of functional and structural constraints on diversity, but less expected of the F gene because of its status as a neutralization and protective antigen, similar to its close ‘relative’ RSV [22]. It has also been suggested that the low diversity in F gene might make a substantial contribution to cross-neutralization and cross-protection between the HMPV subgroups [21]. The relatively high frequency of amino acid diversity in G (and to a lesser extent SH) could be attributable to selective pressure for amino acid change coming from host immunity; and the ability of the protein to tolerate substitutions, which might be due to its proposed extended, unfolded nature [22]. The phylogenetic incongruence observed between whole genome tree and the F and G gene trees, is as reported previously for HMPV [23], and could be attributed to differential rates of evolution, selection pressure or past recombination events [24].

The prevalence of HMPV in hospitalized pediatric population in Kilifi county in coastal Kenya has been reported [7, 25]. However, it is notable that in recent years, HMPV has been detected at low prevalence in Kilifi (unpublished observations from hospital-based pneumonia surveillance). Whether this low prevalence is due to reduced virus transmission, or decreased sensitivity of our HMPV molecular diagnostic assay due to progressive primer/probe mismatches, is yet to be established.

## Conclusion

We present the first full genome sequences of circulating HMPV strains from sub-Saharan Africa. A limitation of our sequencing method, as is common with amplicon sequencing protocols [26, 27], was absent coverage at the 3′ leader and 5′ trailer regions not captured by these primers. Our results demonstrate the application of amplicon sequencing to generate full length HMPV genomes directly from clinical samples. The observed diversity of the individual genes is comparable to that described previously [20–22]. This method and data provide a useful reference for design of local molecular diagnostics and for studies aimed at understanding HMPV epidemiology and evolution in Africa.

## Methods

### HMPV detection and genotype assignment

Nasopharyngeal and oropharyngeal (NP-OP) swab samples were collected from children (1–59 months) hospitalized with pneumonia, four of whom were enrolled in the PERCH study [18] in 2012. The fifth sample was collected from a child enrolled in the routine pneumonia surveillance study at Kilifi County Hospital, Kenya, in 2015. The samples were tested for HMPV by multiplex semi-quantitative real-time reverse transcription PCR (rRT-PCR) assays. The rRT-PCR primers and probes used, cycling conditions and assay set up have been described elsewhere [28, 29]. Fusion (F) and glycoprotein (G) encoding genes of the HMPV positive samples were amplified in a one-step RT-PCR assay (OneStep RT-PCR kit, QIAGEN), as described previously [7]. Partial G or F nucleotide sequences were analyzed by maximum likelihood (ML) phylogenetic trees using IQ-TREE [30], together with reference strains of HMPV subgroups (accession numbers AF371337.2, FJ168779, AY297749, AY530095, JN184401 and AY297748). Five HMPV positive samples from the Kenya and Zambia study sites, belonging to the A2a (*n* = 1), A2b (*n* = 2), B1 (*n* = 1) and B2 (*n* = 1) genetic subgroups based on their G and F gene sequences, were selected for whole genome sequencing. Data on age, sex and clinical assessment information collected at the time of sample collection, for the five selected samples, are shown in Table 3.

### Whole genome sequencing

The sequencing protocol consisted of four steps as follows: (i) primer design, (ii) preparation of primer mixes, (iii) cDNA and PCR (iv) Illumina sequencing and data analysis.

#### Preparation of HMPV Tm48 full genome primers

All human metapneumovirus (HMPV) full genome sequences were retrieved from GenBank (January 2018) using the query (txid162145 (Organism) AND 12000(SLEN):14000(SLEN) NOT patent). Sequence entries with gaps larger than 6 nt were excluded to generate a set of yielding 178 genomes. All possible 23 nt sequences were generated from the genomes dataset and trimmed to a final calculated melting temperature (Tm) of 47.9–49.5 °C. Sequences with homology to rRNA sequences, with GC content outside < 0.3 or > 0.75 or with a single nucleotide fractional content of > 0.6 were discarded. The primer set was then made non-redundant yielding 60,746 potential primers. All potential primers were mapped against the 178 HMPV full genomes and the number of perfect matches (frequency score) was determined as a measure of primer sequence conservation. To select primers, the HMPV genome sequences were divided into amplicons with 222 nt overlap spanning the virus genome. Potential primers that mapped within the terminal 5′ and 3′ 222 nt of each amplicon were identified and the sequence with the highest frequency score was selected, and primers mapping to the reverse bins were reverse complemented. In this manner, 24 primers were selected for each of the 4 HMPV genotype representative genomes (GenBank accession number HMPV A1: AF371337, HMPV A2: FJ168779; HMPV B1: AY525843, and HMPV B2: FJ168778). Because of conservation between genotypes, there was primer redundancy which was removed. The final set of 65 primer sequences, their lengths, calculated Tm, fractional GC content and mapping position on the HMPV genome are presented in Additional file 5: Table S2. The primers were computationally tested against each of the 4 HMPV subgroups. A graphical representation of the primer target sites is presented in Additional file 1: Figure S1.

#### Preparation of primer mixes

Amplification was performed in two reactions. To avoid generating small products from adjacent forward and reverse primers, amplicons were assigned to alternate reactions, with reaction 1 containing primers for amplicons 1,3,5,7,9,11; reaction 2 containing primers for amplicons 2,4,6,8,10,12. Each reverse transcription used Forward Primer Mixes (FPMs) made with 3.0 μl of each reverse primer (100 pmol/μl) plus water to 200 μl to generate a primer concentration of 24 pmol/μl. Two microlitre of the FPM is then used in a 20 μl reverse transcription reaction (2.4 pmol/μl final concentration in reaction or 2.4 μM/primer). For PCR amplification, each amplicon reaction used a separate PCR Primer Mix (PPM) containing 1.5 μl of each 100 pmol/μl forward primer and 1.5 μl of each reverse primer (5.3–5.5 pmol/μl total primer in the PPM). 2 μl PPM was used per 25 μl PCR reaction = 0.5 pmol/μl in reaction (= 500 nM).

#### cDNA synthesis and PCR

Viral nucleic acids were extracted from the original samples using QIAamp Viral RNA Mini kit (QIAGEN). RNA (5 μl) was reverse transcribed into cDNA using SuperScript III (200 U, Invitrogen), RT buffer (1X final concentration, Invitrogen), and 2 μl of FPM in 20 μl reactions. An aliquot of cDNA (5 μl) was amplified in 35 cycles using Phusion High-fidelity PCR kit (New England Biolabs) and 2 μl of PPM in a 25 μl reaction. The PCR mixture was incubated at 98 °C for 30 s, followed by 35 cycles of 98 °C for 10 s, 43 °C for 30 s, and 72 °C for 90s and a final extension of 72 °C for 10 min. Expected PCR products for each amplicon were approximately 1500 bp. PCR products from the two reactions for each sample were pooled for Illumina library preparation.

#### Illumina sequencing and data analysis

Libraries were prepared using Nextera XT kit (Illumina) and pair-end sequencing (2 × 300 base pairs) with the MiSeq Reagent V3 kit (Illumina), following the manufacturer’s instructions. The Nextera enzyme mix was used to simultaneously fragment input DNA and tag with universal adapters in a single tube reaction, followed by 12-cycle PCR reaction for dual indexing. Agencourt AMPure XP beads (Beckman Coulter) were used for all purification steps and libraries were quantified and quality-checked using the Qubit (Thermo Fisher) and Bioanalyzer (Agilent). Adapter trimming, quality filtering, kmer normalization of sequencing reads, de novo assembly, calculation of mean genome coverage was as previously described [31].

### Phylogenetic analyses

**Fig. 5:**
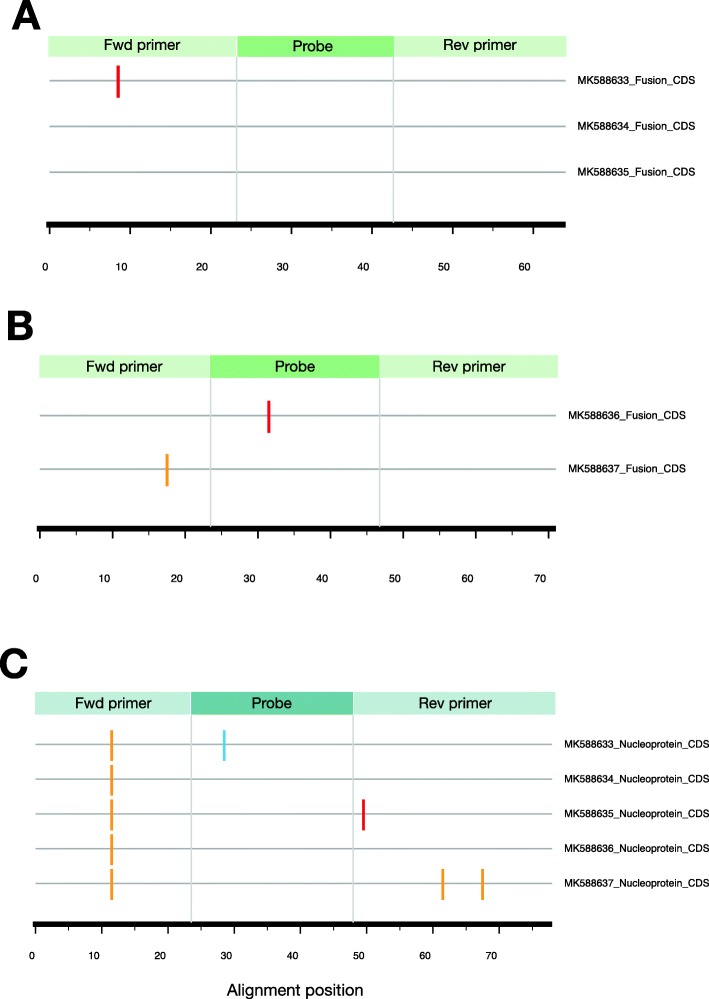
Flow chart diagram depicting a summary of methods applied in this study

A dataset of HMPV genome sequences was retrieved from ViPR in order to infer relationship between HMPV viruses from Kenya and Zambia and viral populations sampled globally. The dataset included 138 sequence entries (> 13,000 nt) that included date (year) and location of sample collection (Additional file 6: Table S3). Sequence alignment was done using MAFFT v.7.221 [32] using the parameters ‘–localpair –maxiterate 1000’. IQ-TREE was used to infer maximum likelihood (ML) trees of the complete genome and individual genes under general time-reversible (GTR) substitution model with gamma-distributed among-site rate heterogeneity. A summary of the methodology outlined here is depicted in Fig. 5.

## Supplementary information

**Additional file 1: Figure S1.** The target positions in HMPV genomes for the set of 65 HMPV primers are presented. Green diamonds indicate forward primers, blue diamonds indicate reverse primers. Primers sites in representative A1, A2, B1 and B2 genomes are shown (HMPV A1: GenBank accession number AF371337, HMPV A2: FJ168779; HMPV B1: AY525843 and HMPV B2: FJ168778).

**Additional file 2: Figure S2.** Average pairwise identity over all pairs in an alignment for every position of the predicted SH glycoprotein amino acid sequences, for HMPV groups A (A) and B (B). The dataset analyzed here included all available genomes (Kenya and Zambia (*n* = 5) plus 138 from other locations globally). Black bars indicate > 50% (> 0.5) average amino acid identity and red bars indicate < 50% (< 0.5) non-identity among sequences. Proposed intracellular (positions 1 to 30), transmembrane (TM, positions 31 to 51), and extracellular (positions 52 to 178 for group A, or 52 to 180 group B) domains are indicated above the plots.

**Additional file 3: Figure S3.** Mid-pointed maximum-likelihood (ML) phylogenetic trees of N gene (A), P gene (B), M gene (C), M2 gene (D), F gene (E), and L gene (F) of viruses from Kenya and Zambia (marked in red), plus 138 other genomes (> 13 kb) retrieved from GenBank. Bootstrap supports (evaluated by 1000 replicates) are indicated at the nodes. Genetic subgroups and lineages, A1, A2a, A2b, B1, and B2, are indicated. Multiple sequence alignment was done using MAFFT v7.221 and the ML phylogeny inferred using GTR + Γ nucleotide substitution model and ultrafast bootstrap approximation in IQ-TREE. The genotype B2 Sabana strain sequence (GenBank accession number HM197719) reported from a wild mountain gorilla in Rwanda is marked in blue.

**Additional file 4: Table S1.** Summaries of HMPV prevalence and incidence as reported in various publications.

**Additional file 5: Table S2.** Primer sequences used in RT-PCR for whole genome amplification. Primer nomenclature is described as follows: AF-11A1 indicates an A reaction, forward primer for amplicon 11, selected for genotype A1 (although it may function against the other 4 genotypes). Also listed are the primer sequence (5′ to 3′) the primer length, the melting temperature (calculated using the Santa Lucia algorithm (https://biopython.org/DIST/docs/api/Bio.SeqUtils.MeltingTemp-module.html) plus 3.7 C) to obtain the Breslauer values [33]) and the fraction of the primer that is G + C [34].

**Additional file 6: Table S3.** Information (GenBank accession, year and country of sample location) of the global sequences used in phylogenetic analyses. (CSV 2 kb)

**Additional file 7: Table S4.** Primer sequences used in the semi-quantitative rRT-PCR for HMPV detection. Note, the F gene-based rRT-PCR assays are different or separate for the two HMPV groups.

## Data Availability

The assembled sequences for the five genomes from Kenya and Zambia are available in the GenBank nucleotide database with accession numbers MK588633 to MK588637, and the raw sequence data are available in the NCBI SRA archive as BioProject PRJNA523302. The datasets and scripts used in analysis are available on Harvard Dataverse site (doi:10.7910/DVN/TVB65V).

## Abbreviations

aa: Amino acid
ARI: Acute respiratory infections
cDNA: Complementary DNA
F: Fusion glycoprotein
G: Attachment glycoprotein
GTR: General time-reversible
HMPV: Human metapneumovirus
L: and large polymerase protein
M: Matrix protein
M2: Transcription enhancer protein
ML: Maximum likelihood
N: Encoding nucleoprotein
NP-OP: Nasopharyngeal and oropharyngeal
ORFs: Open reading frames
P: Phosphoprotein
rRT-PCR: Reverse transcription PCR
RSV: Respiratory syncytial virus
SH: Small hydrophobic protein
Tm: Temperature
WGS: Whole genome sequencing

## References

[1] Schuster JE, Williams JV (2013). Human metapneumovirus. Pediatr Rev.

[2] Adams O, Weis J, Jasinska K, Vogel M, Tenenbaum T (2015). Comparison of human metapneumovirus, respiratory syncytial virus and rhinovirus respiratory tract infections in young children admitted to hospital. J Med Virol.

[3] Papenburg J, Boivin G (2010). The distinguishing features of human metapneumovirus and respiratory syncytial virus. Rev Med Virol.

[4] Groome MJ, Moyes J, Cohen C, Walaza S, Tempia S, Pretorius M (2015). Human metapneumovirus-associated severe acute respiratory illness hospitalisation in HIV-infected and HIV-uninfected south African children and adults. J Clin Virol.

[5] Kenmoe S, Tchendjou P, Vernet MA, Moyo-Tetang S, Mossus T, Njankouo-Ripa M (2016). Viral etiology of severe acute respiratory infections in hospitalized children in Cameroon, 2011-2013. Influenza Other Respi Viruses.

[6] Feikin DR, Njenga MK, Bigogo G, Aura B, Aol G, Audi A (2012). Etiology and incidence of viral and bacterial acute respiratory illness among older children and adults in rural Western Kenya, 2007–2010. PLoS One.

[7] Owor BE, Masankwa GN, Mwango LC, Njeru RW, Agoti CN, Nokes DJ (2016). Human metapneumovirus epidemiological and evolutionary patterns in coastal Kenya, 2007-11. BMC Infect Dis.

[8] Ahmed JA, Katz MA, Auko E, Njenga MK, Weinberg M, Kapella BK (2012). Epidemiology of respiratory viral infections in two long-term refugee camps in Kenya, 2007-2010. BMC Infect Dis.

[9] Bénet T, Sylla M, Messaoudi M, Sánchez Picot V, Telles J-N, Diakite A-A (2015). Etiology and factors associated with pneumonia in children under 5 years of age in Mali: a prospective case-control study. PLoS One.

[10] Jroundi I, Mahraoui C, Benmessaoud R, Moraleda C, Tligui H, Seffar M (2015). A comparison of human metapneumovirus and respiratory syncytial virus WHO-defined severe pneumonia in Moroccan children. Epidemiol Infect.

[11] Agoti CN, Otieno JR, Munywoki PK, Mwihuri AG, Cane PA, Nokes DJ (2015). Local evolutionary patterns of human respiratory syncytial virus derived from whole-genome sequencing. J Virol.

[12] Marston DA, McElhinney LM, Ellis RJ, Horton DL, Wise EL, Leech SL (2013). Next generation sequencing of viral RNA genomes. BMC Genomics.

[13] Dudas G, Bedford T (2019). The ability of single genes vs full genomes to resolve time and space in outbreak analysis. bioRxiv.

[14] Firth C, Lipkin WI (2013). The genomics of emerging pathogens. Annu Rev Genomics Hum Genet.

[15] Gire SK, Goba A, Andersen KG, Sealfon RS, Park DJ, Kanneh L (2014). Genomic surveillance elucidates Ebola virus origin and transmission during the 2014 outbreak. Science (New York, NY).

[16] Mamanova L, Coffey AJ, Scott CE, Kozarewa I, Turner EH, Kumar A (2010). Target-enrichment strategies for next-generation sequencing. Nat Methods.

[17] Li K, Shrivastava S, Brownley A, Katzel D, Bera J, Nguyen AT (2012). Automated degenerate PCR primer design for high-throughput sequencing improves efficiency of viral sequencing. Virol J.

[18] Pollett S, Trovão NS, Tan Y (2018). The transmission dynamics and diversity of human metapneumovirus in Peru. Influenza Other Respi Viruses.

[19] Kamau E, Agoti CN, Lewa CS, Oketch J, Owor BE, Otieno GP, Bett A, Cane PA, Nokes DJ (2017). Recent sequence variation in probe binding site affected detection of respiratory syncytial virus group B by real-time RT- PCR. J Clin Virol.

[20] Moudy RM, Sullender WM, Wertz GW (2004). Variations in intergenic region sequences of human respiratory syncytial virus clinical isolates: analysis of effects on transcriptional regulation. Virology..

[21] Biacchesi S, Skiadopoulos MH, Boivin G, Hanson CT, Murphy BR, Collins PL (2003). Genetic diversity between human metapneumovirus subgroups. Virology..

[22] Piyaratna R, Tollefson SJ, Williams JV (2011). Genomic analysis of four human metapneumovirus prototypes. Virus Res.

[23] Kim JI, Park S, Lee I, Park KS, Kwak EJ, Moon KM (2016). Genome-Wide Analysis of Human Metapneumovirus Evolution. PLoS One.

[24] Sabir JS, Lam TT, Ahmed MM, Li L, Shen Y, Abo-Aba SE (2016). Co-circulation of three camel coronavirus species and recombination of MERS-CoVs in Saudi Arabia. Science (New York, NY).

[25] Berkley JA, Munywoki P, Ngama M, Kazungu S, Abwao J, Bett A, Lassauniére R (2010). Viral etiology of severe pneumonia among Kenyan infants and children. JAMA.

[26] Cruz CD, Torre A, Troncos G, Lambrechts L, Leguia M (2016). Targeted full-genome amplification and sequencing of dengue virus types 1-4 from South America. J Virol Methods.

[27] Smielewska A, Emmott E, Ranellou K, Popay A, Goodfellow I, Jalal H (2018). UK circulating strains of human parainfluenza 3: an amplicon based next generation sequencing method and phylogenetic analysis. Wellcome Open Res.

[28] Hammitt LL, Kazungu S, Welch S, Bett A, Onyango CO, Gunson RN (2011). Added value of an oropharyngeal swab in detection of viruses in children hospitalized with lower respiratory tract infection. J Clin Microbiol.

[29] Driscoll AJ, Karron RA, Morpeth SC, Bhat N, Levine OS, Baggett HC (2017). Standardization of Laboratory Methods for the PERCH Study. Clin Infect Dis.

[30] Schmidt HA, Minh BQ, von Haeseler A, Nguyen L-T (2014). IQ-TREE: a fast and effective stochastic algorithm for estimating maximum-likelihood phylogenies. Mol Biol Evol.

[31] Kamau E, Agoti CN, Ngoi JM, de Laurent ZR, Gitonga J, Cotten M (2019). Complete Genome Sequences of Dengue Virus Type 2 Strains from Kilifi, Kenya. Microbiol Resour Announce.

[32] Katoh K, Standley DM (2013). MAFFT multiple sequence alignment software version 7: improvements in performance and usability. Mol Biol Evol.

[33] Breslauer KJ, Frank R, Blocker H, Marky LA (1986). Predicting DNA duplex stability from the base sequence. Proc Natl Acad Sci U S A.

[34] O'Brien Katherine L., Baggett Henry C., Brooks W. Abdullah, Feikin Daniel R., Hammitt Laura L., Higdon Melissa M., Howie Stephen R.C., Deloria Knoll Maria, Kotloff Karen L., Levine Orin S., Madhi Shabir A., Murdoch David R., Prosperi Christine, Scott J. Anthony G., Shi Qiyuan, Thea Donald M., Wu Zhenke, Zeger Scott L., Adrian Peter V., Akarasewi Pasakorn, Anderson Trevor P., Antonio Martin, Awori Juliet O., Baillie Vicky L., Bunthi Charatdao, Chipeta James, Chisti Mohammod Jobayer, Crawley Jane, DeLuca Andrea N., Driscoll Amanda J., Ebruke Bernard E., Endtz Hubert P., Fancourt Nicholas, Fu Wei, Goswami Doli, Groome Michelle J., Haddix Meredith, Hossain Lokman, Jahan Yasmin, Kagucia E. Wangeci, Kamau Alice, Karron Ruth A., Kazungu Sidi, Kourouma Nana, Kuwanda Locadiah, Kwenda Geoffrey, Li Mengying, Machuka Eunice M., Mackenzie Grant, Mahomed Nasreen, Maloney Susan A., McLellan Jessica L., Mitchell Joanne L., Moore David P., Morpeth Susan C., Mudau Azwifarwi, Mwananyanda Lawrence, Mwansa James, Silaba Ominde Micah, Onwuchekwa Uma, Park Daniel E., Rhodes Julia, Sawatwong Pongpun, Seidenberg Phil, Shamsul Arifin, Simões Eric A.F., Sissoko Seydou, Wa Somwe Somwe, Sow Samba O., Sylla Mamadou, Tamboura Boubou, Tapia Milagritos D., Thamthitiwat Somsak, Toure Aliou, Watson Nora L., Zaman Khalequ, Zaman Syed M.A. (2019). Causes of severe pneumonia requiring hospital admission in children without HIV infection from Africa and Asia: the PERCH multi-country case-control study. The Lancet.

