# Correction to: Whole genome sequencing and phylogenetic analysis of human metapneumovirus strains from Kenya and Zambia

**DOI:** 10.1186/s12864-020-6498-z

**Published:** 2020-01-28

**Authors:** Everlyn Kamau, John W. Oketch, Zaydah R. de Laurent, My V. T. Phan, Charles N. Agoti, D. James Nokes, Matthew Cotten

**Affiliations:** 1 KEMRI-Wellcome Trust Research Programme, Kilifi, Kenya; 2 Department of Viroscience, Erasmus MC, Rotterdam, The Netherlands; 3 School of Life Sciences and Zeeman Institute, University of Warwick, Coventry, UK; 4 MRC/ UVRI & LSHTM Uganda Research Unit, Entebbe, Uganda; 5 MRC-University of Glasgow Centre for Virus Research, Glasgow, UK

**Correction to: BMC Genomics (2020) 21:5**

**https://doi.org/10.1186/s12864-019-6400-z**

Following the publication of this article [1], it was noted that due to a typesetting error the figure legends were paired incorrectly. The figure legends for Figs. 1, 2, 3, 4 and 5 were wrongly given as captions for Figs. 2, 3, 4, 5 and 1 respectively.

**Fig. 1:**
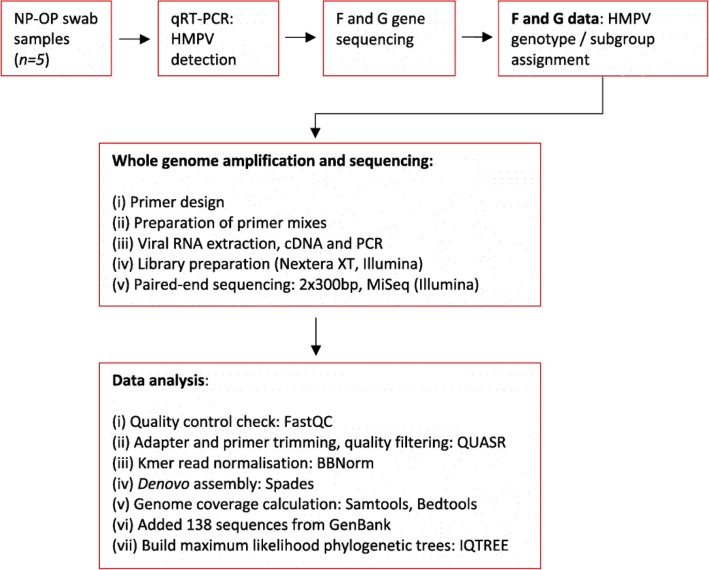
Flow chart diagram depicting a summary of methods applied in this study

**Fig. 2:**
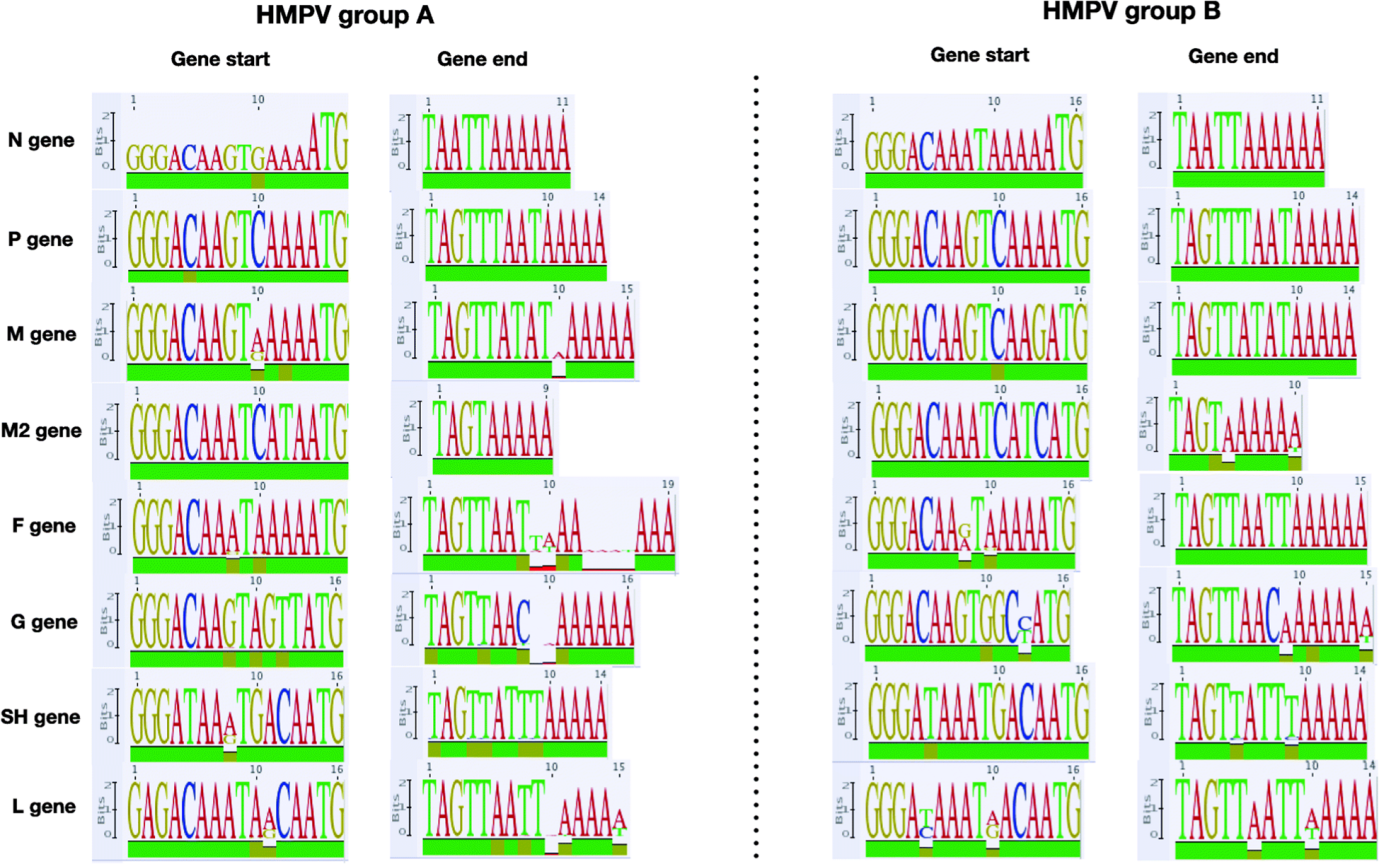
Consensus nucleotide sequences of putative gene-start (13 nucleotides upstream of ATG codon) and gene-end signals (6–16 nucleotides from Stop codon) visualized as sequence logos, for HMPV group (**a**) and (**b**). The height of each character in the sequence logo plots is proportional to its relative frequency. The green color on the bar at the bottom of the consensus sequence logo indicates 100% average pairwise identity, brown indicates at least 30 to < 100% identity and red indicates < 30% identity

**Fig. 3:**
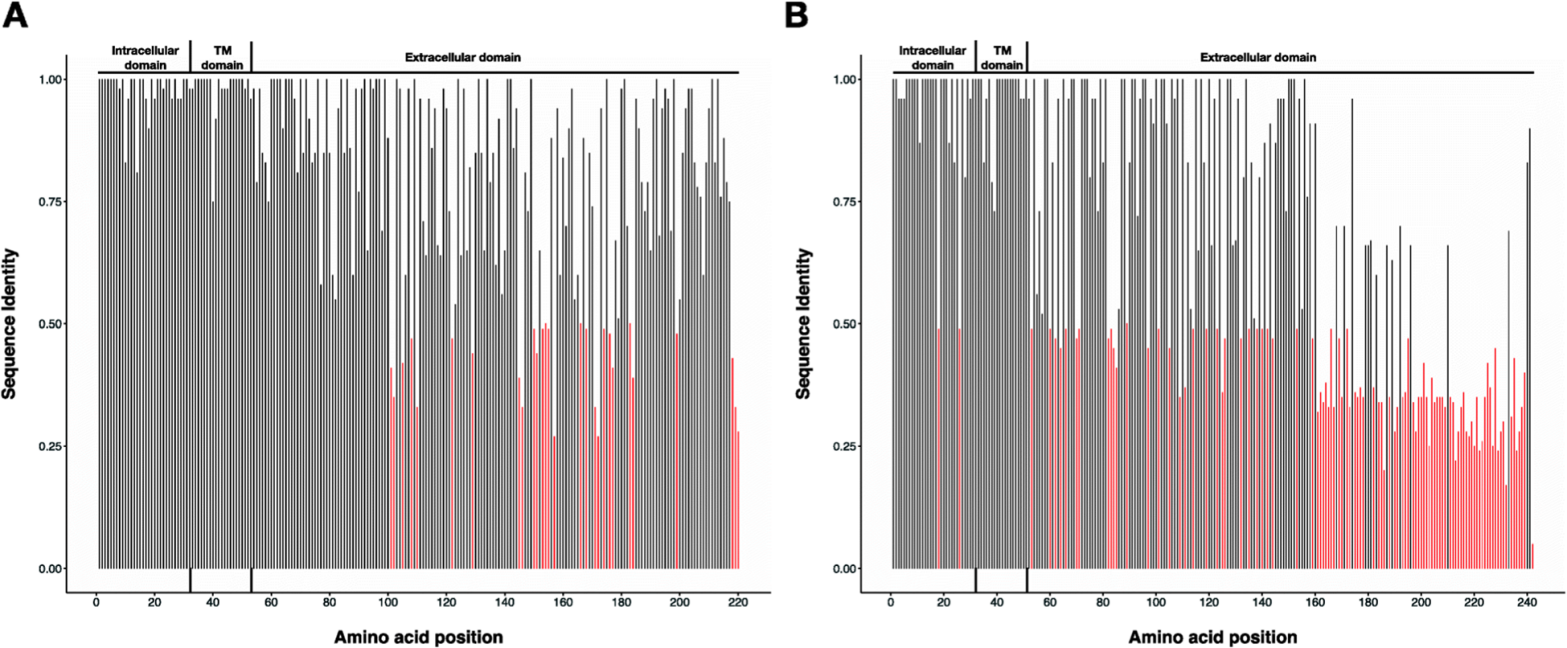
Average pairwise identity over all pairs in an alignment for every position of the predicted G glycoprotein amino acid sequences, for HMPV groups (**a**) and (**b**). The dataset analyzed here included all available genomes (Kenya and Zambia (*n* = 5) plus 138 from other locations globally). The average pairwise identities were calculated in Geneious R8.1.5. Black bars indicate > 50% (> 0.5) average amino acid identity and red bars indicate < 50% (< 0.5) non-identity among sequences. Proposed intracellular (positions 1 to 32), transmembrane (TM, positions 33 to 51), and extracellular (positions 52 to 220 for group (**a**), 52 to 242 for group (**b**) domains are indicated above the plots

**Fig. 4:**
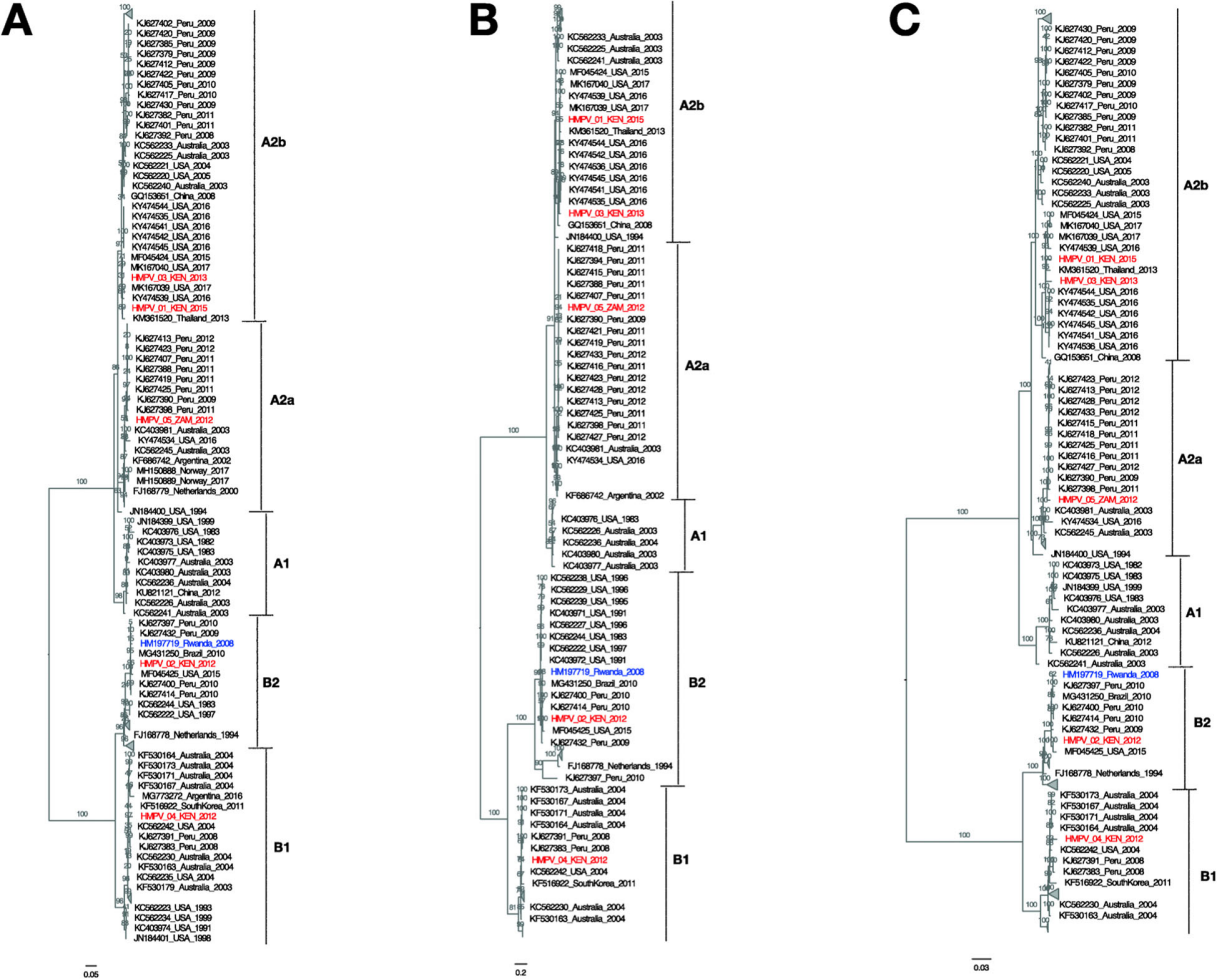
Mid-pointed maximum-likelihood (ML) phylogenetic trees of SH glycoprotein gene (**a**) G glycoprotein gene (**b**) and the full-length genome sequences (**c**) of viruses from Kenya and Zambia (marked in red), plus 138 other sequences (> 13 kb) retrieved from GenBank (Additional Table 3). Bootstrap support values (evaluated by 1000 replicates) are indicated along the branches. Genetic subgroups A1, A2a, A2b, B1, and B2, are indicated. Multiple sequence alignment was done using MAFFT and the ML phylogeny inferred using GTR + Γ nucleotide substitution model and ultrafast bootstrap approximation in IQ-TREE. The genotype B2 Sabana strain sequence (GenBank accession number HM197719) reported from a wild mountain gorilla in Rwanda is marked in blue. The scaled bar indicates nucleotide substitutions per site

**Fig. 5:**
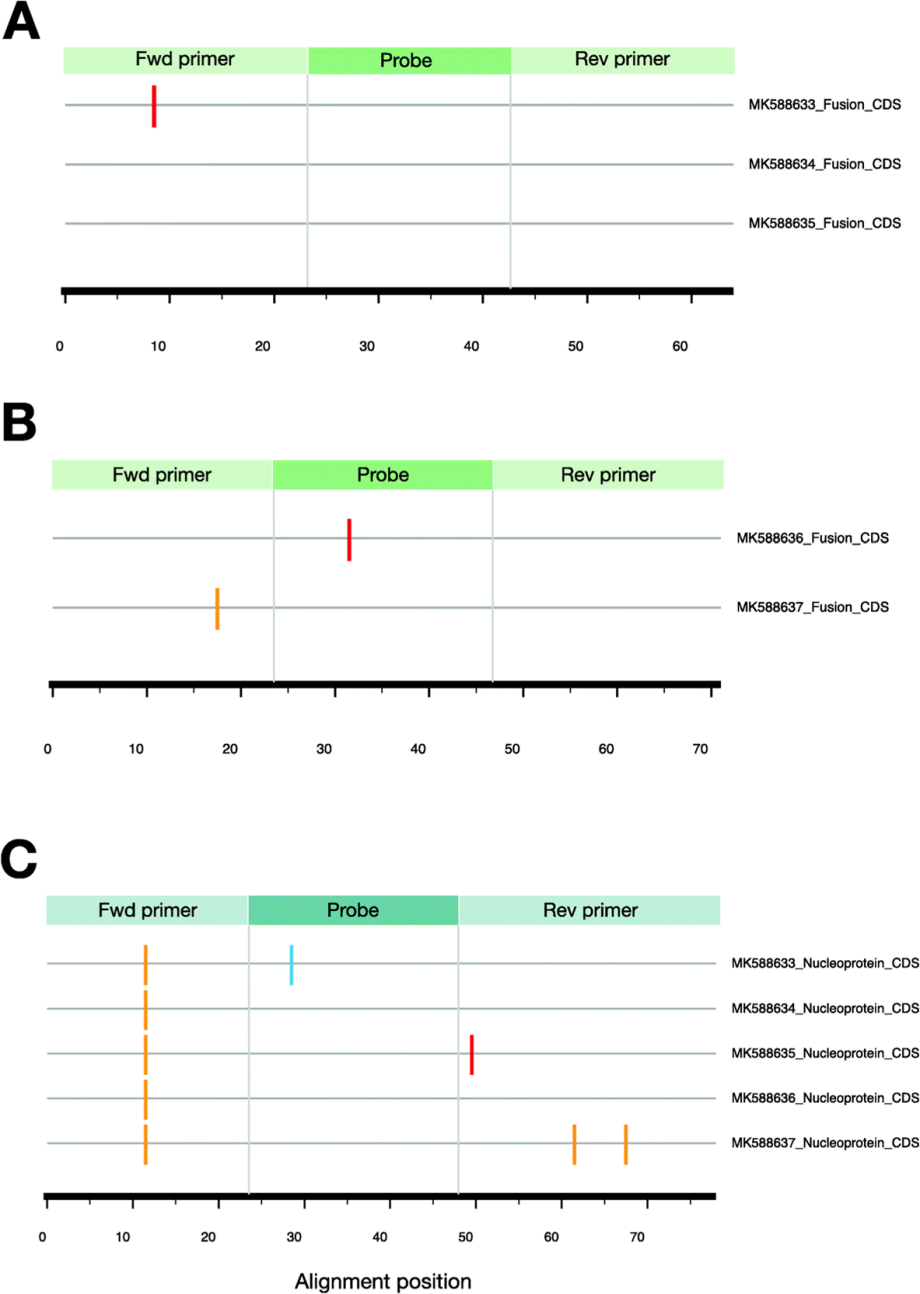
Mismatches between the rRT-PCR diagnostic primers and probes and their expected binding sites in the five genomes from Kenya and Zambia. ‘Fwd primer’ = Forward primer and ‘Rev primer’ = Reverse primer. Two rRT-PCR assays were used for HMPV detection. The colored bars in the figure indicate nucleotide differences (mismatches) between (**a**) three HMPV-A genomes and HMPV-A specific primers and probes targeting fusion gene, (**b**) two HMPV-B genomes and HMPV-B specific primers and probes also targeting fusion gene, and (**c**) all five genomes reported here and specific primers and probes targeting nucleoprotein gene. The sequences of the rRT-PCR primers and probes checked against the African HMPV genomes are listed in Additional file 7: Table S4

The correct figures and captions have been included in this Correction, and the original article has been corrected.
