# Supplementary figures and images for: Whole genome sequencing and phylogenetic analysis of human metapneumovirus strains from Kenya and Zambia
Source: BMC Genomics. 2020 Jan 2;21:5. doi: 10.1186/s12864-019-6400-z (PMC6941262; doi:10.1186/s12864-019-6400-z)

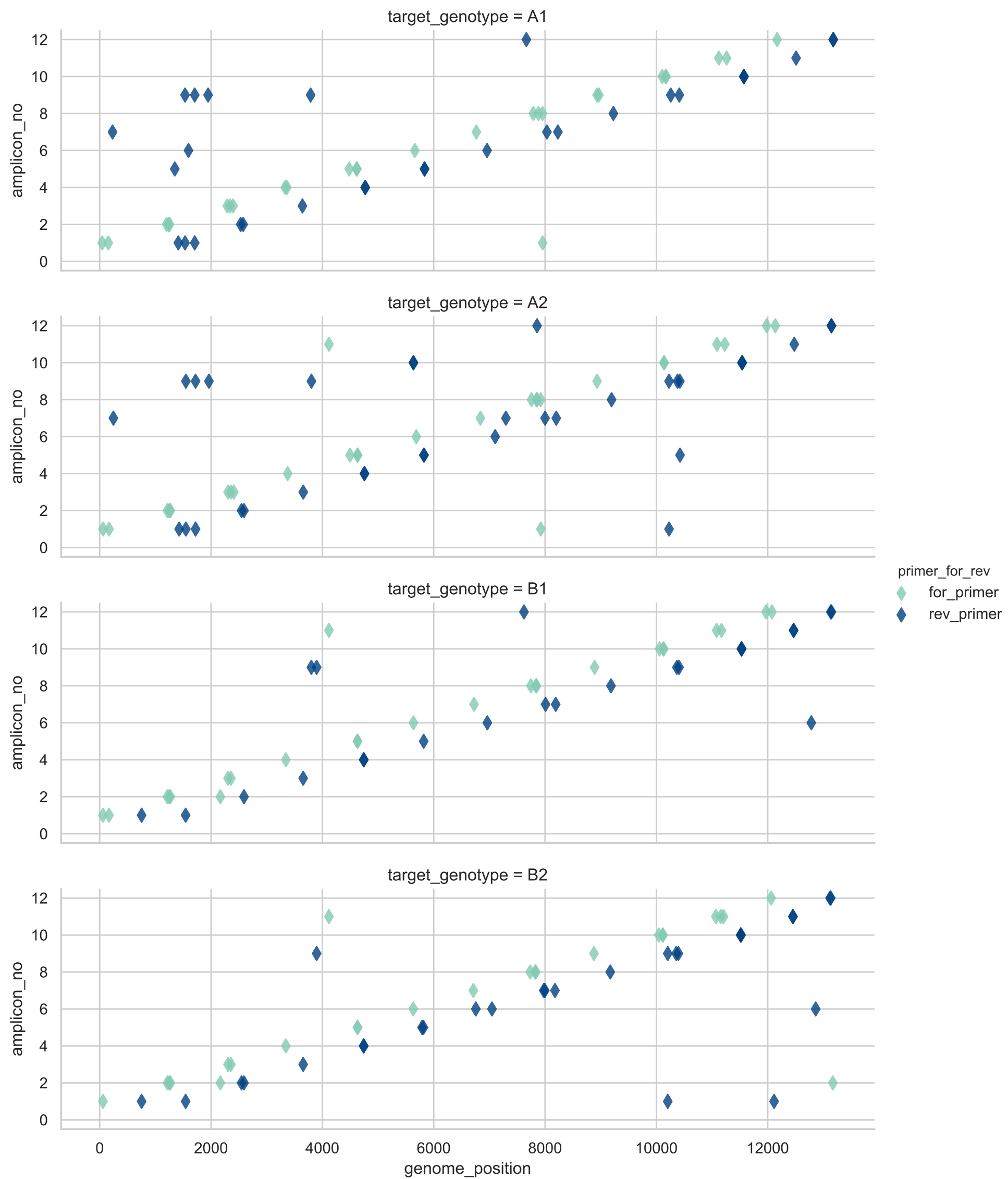

Supplement: Supplementary file 1 — Additional file 1: Figure S1. The target positions in HMPV genomes for the set of 65 HMPV primers are presented. Green diamonds indicate forward primers, blue diamonds indicate reverse primers. Primers sites in representative A1, A2, B1 and B2 genomes are shown (HMPV A1: GenBank accession number AF371337, HMPV A2: FJ168779; HMPV B1: AY525843 and HMPV B2: FJ168778). [file 12864_2019_6400_MOESM1_ESM.pdf]

**A**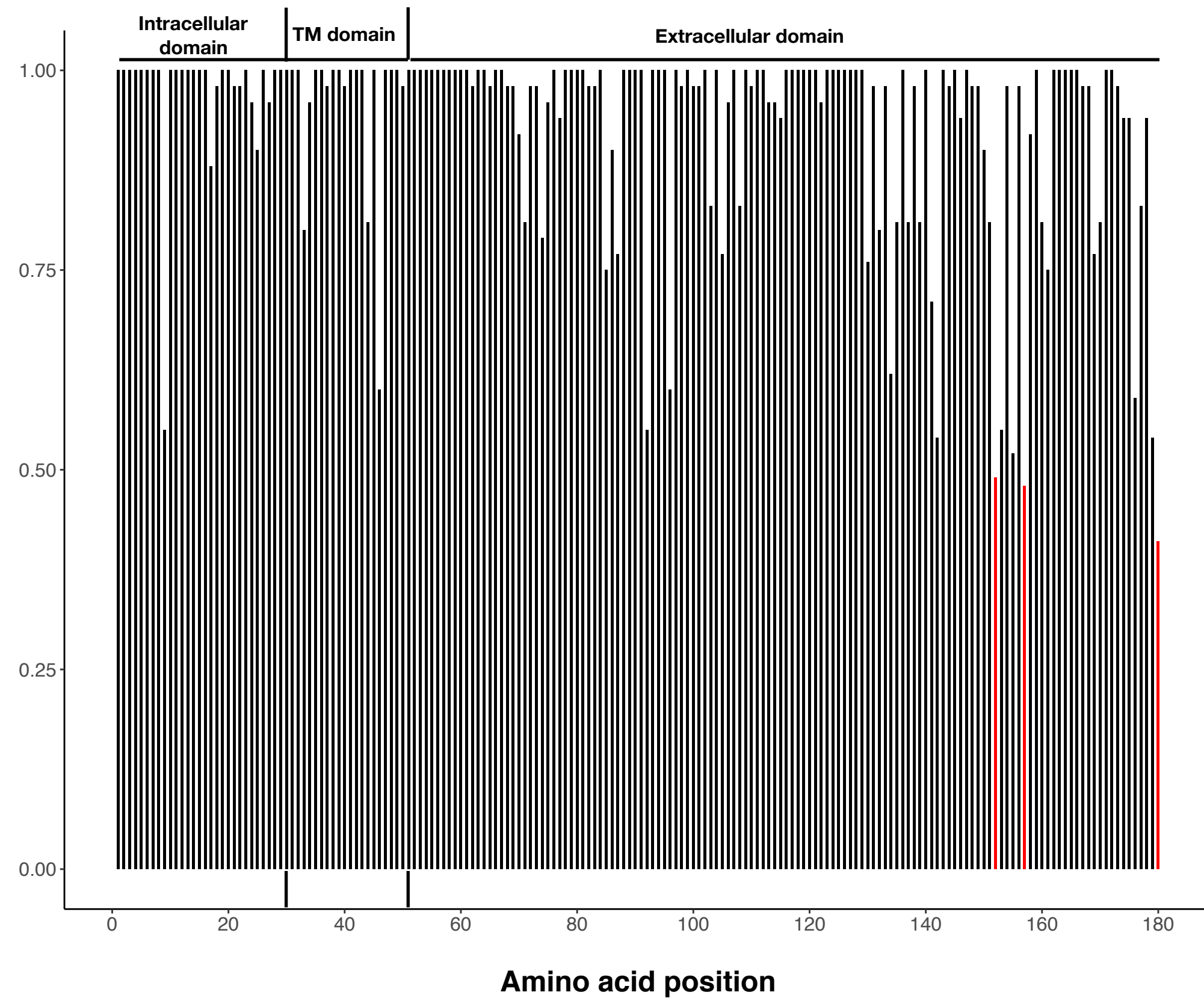**B**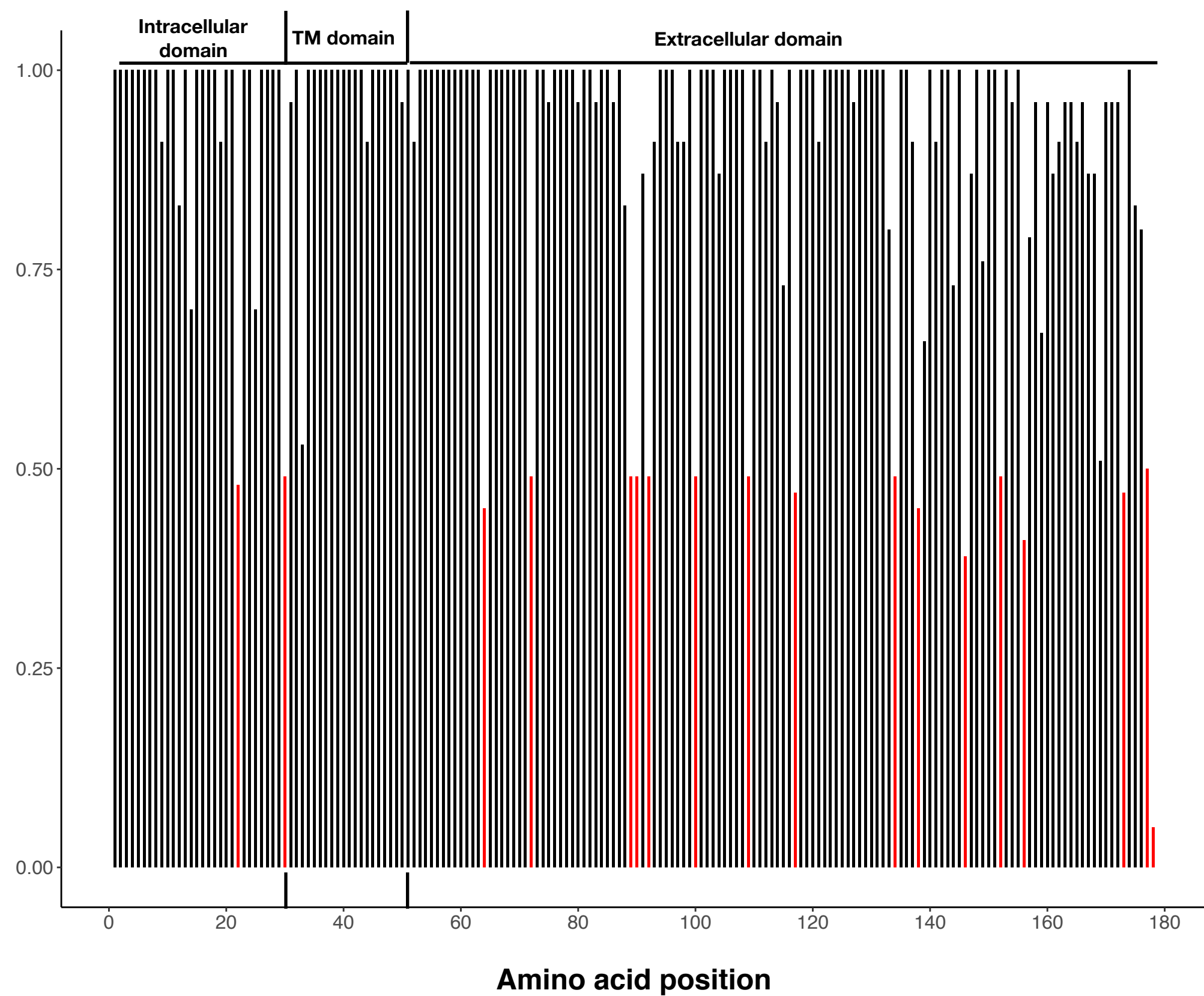

Supplement: Supplementary file 2 — Additional file 2: Figure S2. Average pairwise identity over all pairs in an alignment for every position of the predicted SH glycoprotein amino acid sequences, for HMPV groups A (A) and B (B). The dataset analyzed here included all available genomes (Kenya and Zambia (n = 5) plus 138 from other locations globally). Black bars indicate > 50% (> 0.5) average amino acid identity and red bars indicate < 50% (< 0.5) non-identity among sequences. Proposed intracellular (positions 1 to 30), transmembrane (TM, positions 31 to 51), and extracellular (positions 52 to 178 for group A, or 52 to 180 group B) domains are indicated above the plots. [file 12864_2019_6400_MOESM2_ESM.pdf]

**A**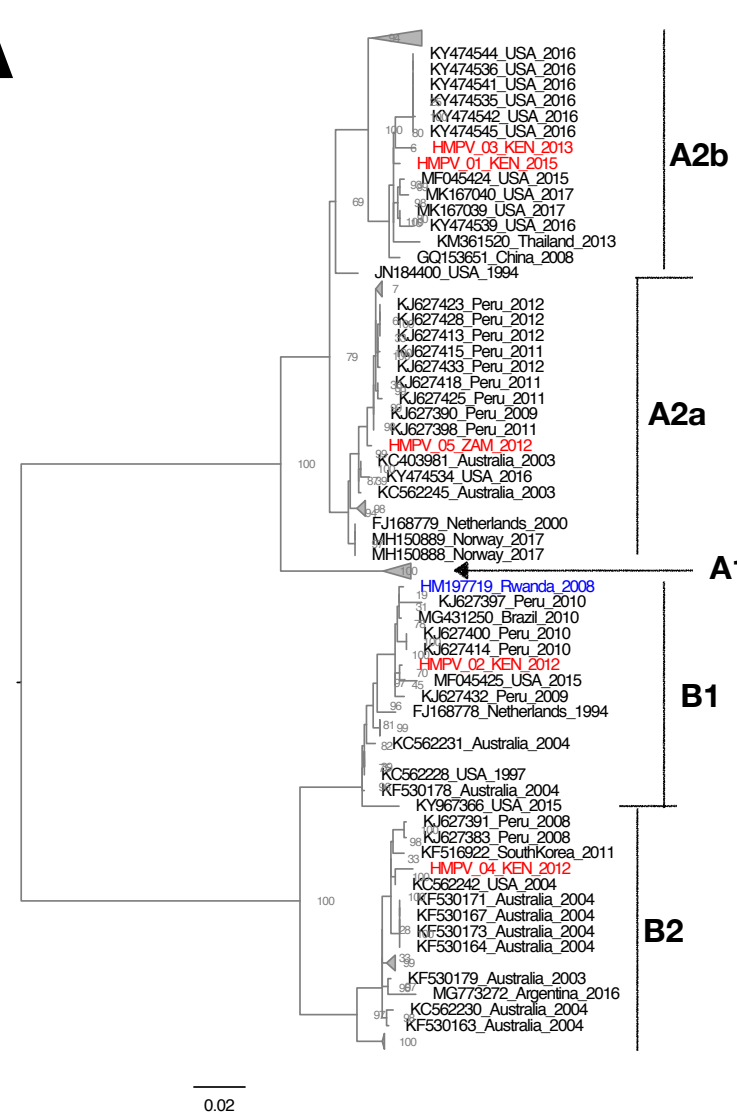**B**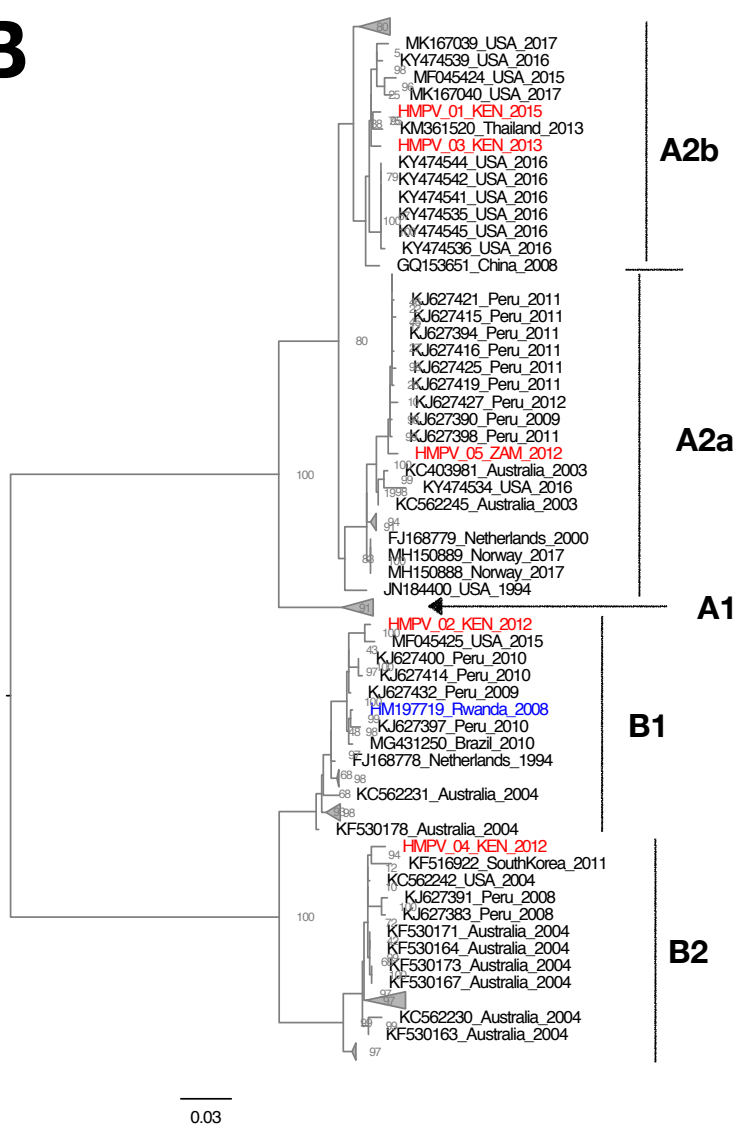**C**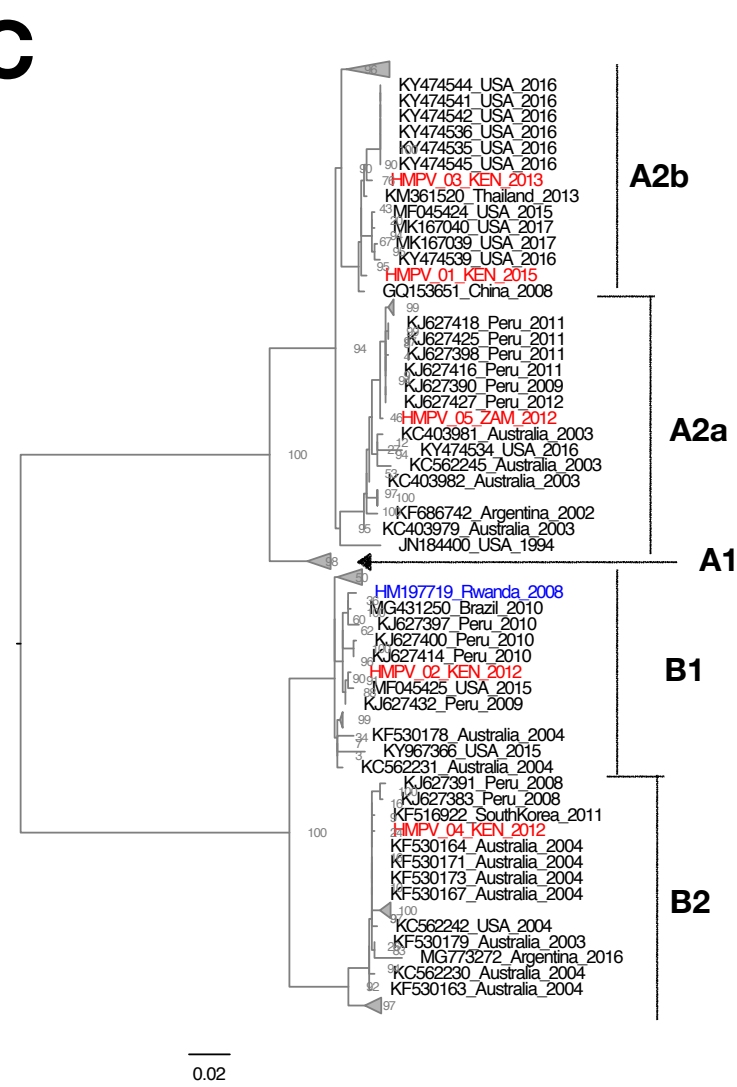**D**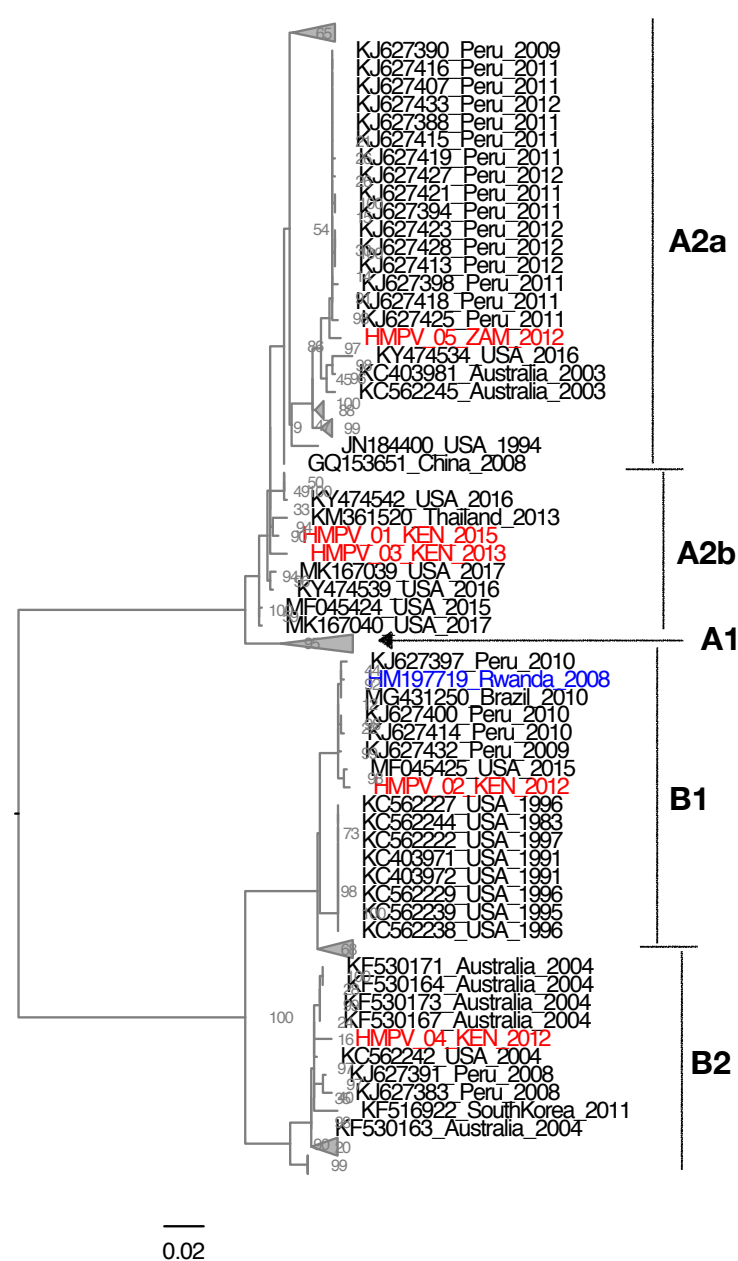**E**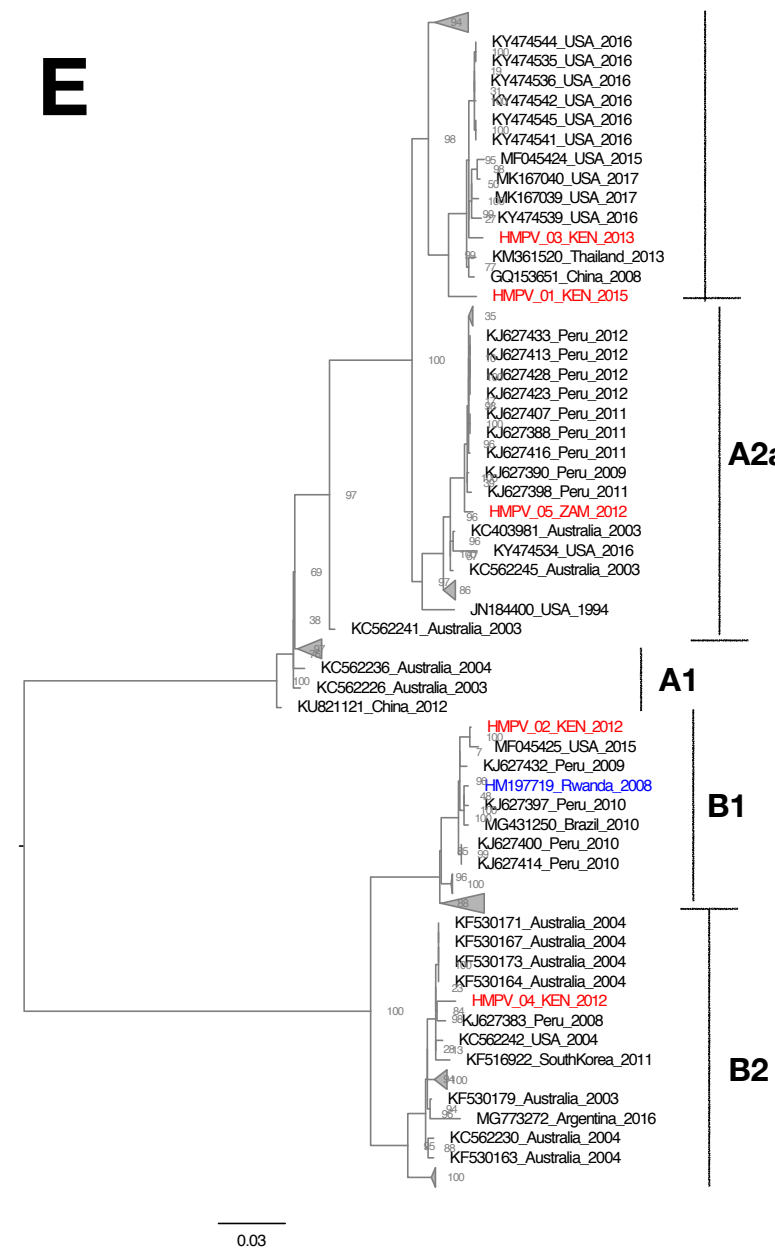**F**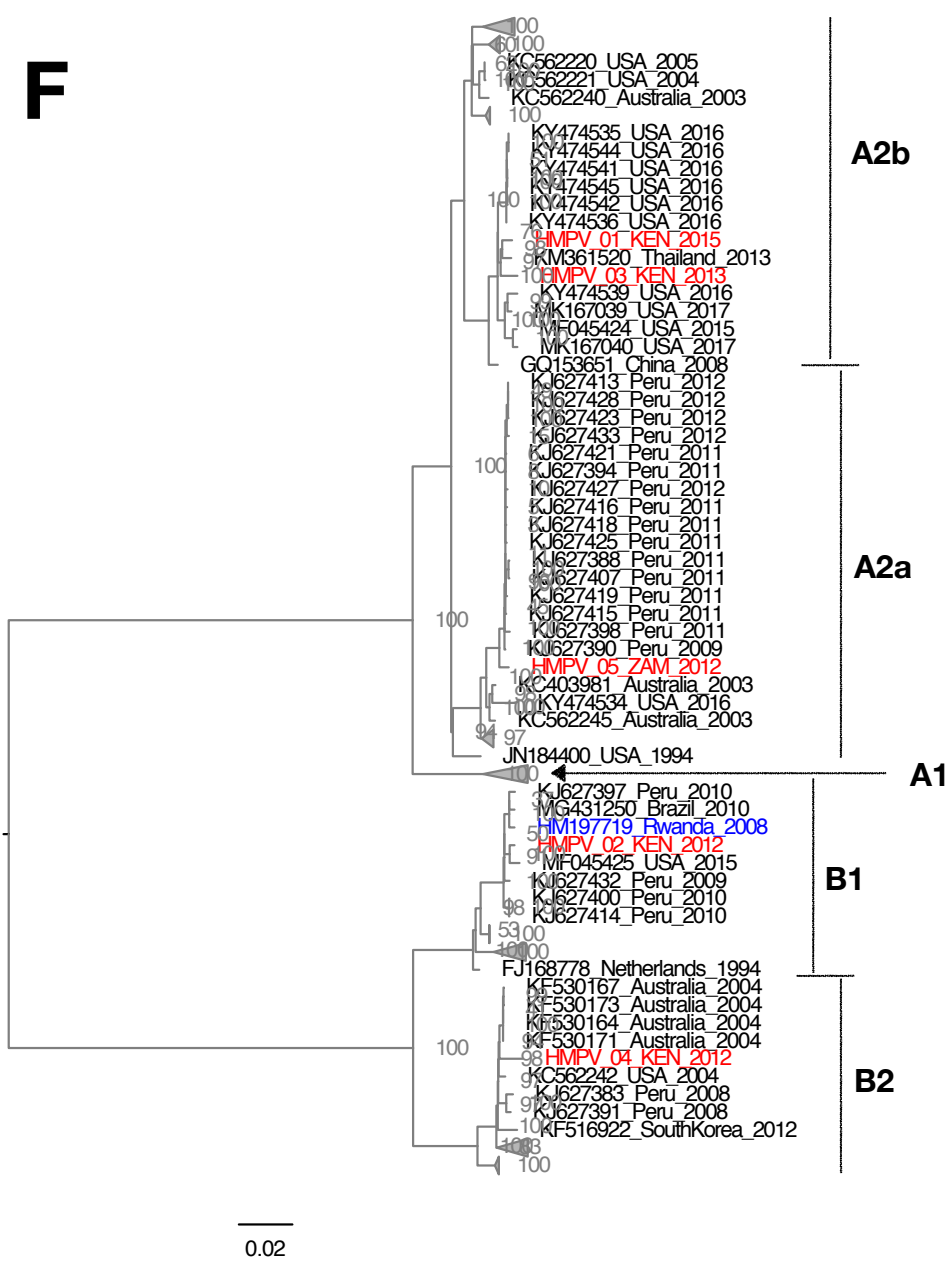

Supplement: Supplementary file 3 — Additional file 3: Figure S3. Mid-pointed maximum-likelihood (ML) phylogenetic trees of N gene (A), P gene (B), M gene (C), M2 gene (D), F gene (E), and L gene (F) of viruses from Kenya and Zambia (marked in red), plus 138 other genomes (> 13 kb) retrieved from GenBank. Bootstrap supports (evaluated by 1000 replicates) are indicated at the nodes. Genetic subgroups and lineages, A1, A2a, A2b, B1, and B2, are indicated. Multiple sequence alignment was done using MAFFT v7.221 and the ML phylogeny inferred using GTR + Γ nucleotide substitution model and ultrafast bootstrap approximation in IQ-TREE. The genotype B2 Sabana strain sequence (GenBank accession number HM197719) reported from a wild mountain gorilla in Rwanda is marked in blue. [file 12864_2019_6400_MOESM3_ESM.pdf]
