# Supplementary material for: Whole genome sequencing and phylogenetic analysis of human metapneumovirus strains from Kenya and Zambia
Source: BMC Genomics. 2020 Jan 2;21:5. doi: 10.1186/s12864-019-6400-z (PMC6941262; doi:10.1186/s12864-019-6400-z)
Supplement: Supplementary file 4 — Additional file 4: Table S1. Summaries of HMPV prevalence and incidence as reported in various publications. [file 12864_2019_6400_MOESM4_ESM.docx]

**Table S1** Summaries of HMPV prevalence and incidence as reported in various publications.

| **Country** | **Prevalence / Incidence** | **Reference** |
| --- | --- | --- |
| US | HMPV was associated with 5% to 13% of pediatric hospitalizations for respiratory illness | [[35](#_ENREF_31)] |
| Guatemala | Annual incidence of hospitalized HMPV-ARI was estimated as 102/100000 children aged <5 years, 2.6/100000 persons aged 5-17 years, and 2.6/100 000 persons aged ≥ 18 years | [[3](#_ENREF_32)6] |
| Norway | Average annual hospitalization rates due to HMPV was estimated at 1.9/1000 in Norwegian children <5 years old with lower RTI | [[3](#_ENREF_33)7] |
| Jordan | HMPV was associated with 8.6% of cases in children less than 2 years | [[3](#_ENREF_34)8] |
| Germany | 11.9% of hospitalized children with bronchitis, pneumonia or pharyngitis were positive for HMPV | [[3](#_ENREF_35)9] |
| Argentina | Proportion of HMPV infections in hospitalized children ranged from 18% in those less than 6 months of age and 5% in children under 5 years | [[40](#_ENREF_36)] |
| India | Attributable adjusted etiologic fraction for HMPV in acute lower respiratory infections in children under-ten in rural Ballabgarh, northern India, 2012-14 was 86% (95% CI 71-93) | [41] |

**References**

35. Jain S, Williams DJ, Arnold SR, Ampofo K, Bramley AM, Reed C, et al. Community-acquired pneumonia requiring hospitalization among U.S.children. N Engl J Med. 2015;372(9):835–45.

36. McCracken JP, Arvelo W, Ortiz J, Reyes L, Gray J, Estevez A, et al. Comparative epidemiology of human metapneumovirus- and respiratory syncytial virus-associated hospitalizations in Guatemala. Influenza Other Respi Viruses. 2014;8(4):414–21.

37. Moe N, Stenseng IH, Krokstad S, Christensen A, Skanke LH, Risnes KR, et al. The

burden of human Metapneumovirus and respiratory syncytial virus infections in hospitalized Norwegian children. J Infect Dis. 2017;216(1):110–6.

38. Schuster JE, Khuri-Bulos N, Faouri S, Shehabi A, Johnson M, Wang L, et al. Human Metapneumovirus infection in Jordanian children: epidemiology and risk factors for severe disease. Pediatr Infect Dis J. 2015;34(12):1335–41.

39. Reiche J, Jacobsen S, Neubauer K, Hafemann S, Nitsche A, Milde J, et al. Human metapneumovirus: insights from a ten-year molecular and epidemiological analysis in Germany. PLoS One. 2014;9(2):e88342.

40. Marcone DN, Durand LO, Azziz-Baumgartner E, Vidaurreta S, Ekstrom J,

Carballal G, et al. Incidence of viral respiratory infections in a prospective cohort of outpatient and hospitalized children aged </=5 years and its associated cost in Buenos Aires, Argentina. BMC Infect Dis. 2015;15:447.

41. Krishnan A, Kumar R, Broor S, Gopal G, Saha S, Amarchand R, et al.Epidemiology of viral acute lower respiratory infections in a communitybased cohort of rural north Indian children. J Glob Health. 2019;9(1):010433.
